# Commentary on: Productivity and Efficiency of a Department Resident Aesthetic Plastic Surgery Clinic

**DOI:** 10.1093/asjof/ojad006

**Published:** 2023-01-25

**Authors:** Nicholas R Sinclair

**Affiliations:** Department of Plastic Surgery, University of Texas Southwestern, Dallas, TX, USA

I commend the authors on furthering the body of literature assessing the safety of resident aesthetic clinics (RACs) (Video).^1^ Millions of patients each year undergo aesthetic plastic surgery, and this number is rising.^2^ Accordingly, the Accreditation Council for Graduate Medical Education has increased the required number of aesthetic cases to fulfil residency training.^3^ RACs are often considered the most useful form of aesthetic surgery training.^4-6^ This retrospective review^1^ demonstrates the utility and safety of an RAC, a finding which is in line with previous studies and systematic reviews.^7,8^

While safety is an understandable starting point of an RAC, it is not the only metric by which competency should be evaluated. Measurements of clinical outcomes are critical to the assessment of an aesthetic surgery practice. These types of studies, unfortunately, are lacking with regards to RACs. The authors do provide a low revision rate as a means of assessing patient satisfaction. However, as the authors point out, the short follow-up time (median 29 days; range, 4-305 days) is a limitation in truly estimating patient satisfaction and the eventual need for revisions. I look forward to future studies with a longer follow-up time.

As the aesthetic surgery marketplace has expanded, plastic surgeons have often touted our rigorous training as a differentiator from other aesthetic providers. To better understand and improve aesthetic surgery training, future studies of RACs should focus on outcome measurements through validated means.

## Supplementary Material

ojad006_Supplementary_Data
